# Transcutaneous pollinosis immunotherapy using a solid‐in‐oil nanodispersion system carrying T cell epitope peptide and R848

**DOI:** 10.1002/btm2.10048

**Published:** 2017-02-03

**Authors:** Momoko Kitaoka, Ayaka Naritomi, Yoshinori Kawabe, Masamichi Kamihira, Noriho Kamiya, Masahiro Goto

**Affiliations:** ^1^ Dept. of Applied Chemistry Kyushu University 744 Moto‐oka Fukuoka 819‐0395 Japan; ^2^ Dept. of Chemical Engineering Kyushu University 744 Moto‐oka Fukuoka 819‐0395 Japan; ^3^ Advanced Transdermal Drug Delivery Center Kyushu University 744 Moto‐oka Fukuoka 819‐0395 Japan; ^4^ Center for Future Chemistry Kyushu University 744 Moto‐oka Fukuoka 819‐0395 Japan

**Keywords:** immunotherapy, R848, Resiquimod, solid‐in‐oil nanodispersion, T cell epitope, transcutaneous drug delivery

## Abstract

Antigen‐specific immunotherapy is the only curative approach for the treatment of allergic diseases such as Japanese cedar pollinosis. Immunotherapy using a T cell epitope vaccine in combination with the adjuvant R848 is of particular interest as a safe and effective approach to treat allergic diseases. Herein, we propose a simple and easy to handle vaccine administration method using the original solid‐in‐oil (S/O) nanodispersion system that permeates through the skin. The S/O nanodispersion system is composed of nanoparticles of hydrophilic molecules surrounded with hydrophobic surfactants that are dispersed in an oil vehicle. The system has potential to carry and deliver both hydrophilic and hydrophobic bioactives. Hydrophilic T cell epitope peptide was efficiently delivered through mouse skin using the S/O nanodispersion system and lowered antigen‐specific IgE levels in pollinosis model mice. Addition of the hydrophobic adju1vant R848 significantly lowered the antibody secretion and shifted the Th1/Th2‐balance toward Th1‐type immunity in the model mice, showing the potential to alleviate Japanese cedar pollinosis.

**Abbreviations**
5‐CF: 5‐carboxyfluorescein
DLS: dynamic light scattering
IPM: isopropyl myristate
PBS: phosphate buffered saline
Rho‐DOPE: Rhodamine‐dioleyl phosphatidylethanolamine
s.c. injection: subcutaneous injection
S/O nanodispersion: solid‐in‐oil nanodispersion
Th1 cell: type 1 helper T cell
Th2 cell: type 2 helper T cell
TLR: toll like receptor

## 1 Introduction

Vaccines have contributed to the decrease in the rate of infectious diseases since their introduction more than two centuries ago.^1^ Recent research has revealed vaccines are useful treatments for a number of immune‐related diseases including autoimmune diseases, cancers, and allergies.^2^, ^3^, ^4^ Allergic diseases have been conventionally classified into four types (type I, II, III, and IV) according to Gell and Coombs,^5^ although many exceptions have recently been found that do not fit these clasifications.^6^, ^7^ Type I (immediate‐type) allergy, such as pollinosis, cat allergy, house dust mite allergy, or allergic asthma, is caused by activation of type 2 helper T (Th2) cells and induction of IgE antibodies from B cells. The representative symptoms of type I allergy are rhinitis, conjunctivitis, pruritus, asthma, and lowered blood pressure. These allergic reactions are triggered by the binding of antigen molecules to IgE on mast cells, and the symptoms appear within 5–15 min from the time of contact with the antigens. Type I allergy holds risks of severe symptoms such as anaphylaxis^8^ and the only curative treatments of this type of allergy are immunotherapies using whole antigen molecules, antigen derivatives, or T cell epitopes.^9^, ^10^

T cell epitopes are short specific regions of the antigen molecules (usually 10–20 amino acids) that are recognized by mammalian T cells.^11^ Researches have recently suggested that several mechanisms are involved in peptide immunotherapy using T cell epitopes, including activation of regulatory T cells, induction of T cell anergy and deletion of allergen‐specific Th2 cells.^12^ Type 1 helper T (Th1) cell‐dominant immunity is observed in the sera from patients that have been treated with T cell epitopes. Stimulation of IgG antibody secretions were also observed.^13^ Serious adverse events are a problem using whole antigen molecules in immunotherapy. T cell epitopes do not bind to IgE and the likelihood of serious adverse events are expected to be low during T cell epitopetherapy. Several recent reports demonstrated that a conjugated peptide of seven T cell epitopes derived from Japanese cedar (*Cryptomeria japonica*) pollen allergen (Cry j 1 and 2) (7Crp) has potential to alleviate allergic symptoms in murine models.^14^, ^15^ Japanese cedar pollinosis is becoming a severe problem in Japan. One quarter of Japanese population is assumed to suffer from the pollinosis according to a survey in 2010 and its prevalence is thought to be increasing.^16^, ^17^ Therefore, development of an effective therapy is a high priority.

**Figure 1:**
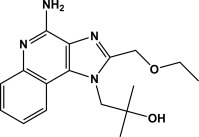
Chemical structure of R848 (Resiquimod)

Conventional immunotherapy of type I allergy requires lengthy treatments from several months to years of vaccine inoculations administered by subcutaneous (s.c.) injection or by the sublingual mucosal route. The pain associated with injections lowers patient compliance using the s.c. route. Similarly, adverse events resulting from sublingual immunotherapy (i.e., local swelling, itchiness, and gastrointestinal inflammation) possibly raise the therapy discontinuation rate. Administration of vaccines via a patch is advantageous because of the ease of handling and painless application.^18^ Vaccines delivered by the transcutaneous route encounter abundant immune‐related cells in the skin,^19^, ^20^, ^21^ bypassing metabolic pathways.^22^ The outermost layer of the skin is hydrophobic and functions as a barrier to prevent intrusion of extraneous molecules and organisms, making passive diffusion permeability of peptides and proteins quite low.^23^, ^24^ To address this issue, we have previously used transcutaneous administration of an arginine‐modified 7Crp, 7CrpR, by a unique solid‐in‐oil (S/O) nanodispersion drug carrier system.^25^ The S/O nanodispersion was prepared by lyophilizing a water‐in‐oil (W/O) emulsions consists of 7CrpR in water and a surfactant in vaporous organic solvents to give a solid paste that was redispersed in an oil vehicle. Dispersing the nanosized solid peptides in an oil vehicle ensured they permeated efficiently across the hydrophobic layer,^26^, ^27^ although the therapeutic effect of 7CrpR was unsatisfactory. In this study, we introduce a hydrophobic adjuvant, R848 (Resiquimod, Figure 1), to the 7CrpR S/O nanodispersion system. R848 is a toll like receptor (TLR) 7/8 agonist that acts as an immune response modifier, which shifts the Th1/Th2 immune balance toward Th1‐dominant immunity, and is known to alleviate allergic symptoms.^28^

## 2 Experimental

### 2.1 Animals

Female BALB/c mice (7‐week‐old) were purchased from Kyudo (Saga, Japan) a week prior to experimentation, and housed at a controlled temperature (23 °C) with a 12 h light/dark cycle. Animal experiments were carried out with approval of the Ethics Committee for Animal Experiments in Kyushu University, and in accordance with the Guide for the Care and Use of Laboratory Animals from Science Council of Japan.

### 2.2 Materials

R848 was purchased from Enzo life science (Farmingdale, NY). Cyclohexane and Rhodamine‐dioleyl phosphatidylethanolamine (Rho‐DOPE) were purchased from Wako Pure Chemical Industries (Kyoto, Japan) and Avanti Polar Lipids (Alabaster, AL), respectively. Isopropyl myristate (IPM) and 5‐carboxyfluorescein (5‐CF) *N*‐succinimidyl ester were purchased from Tokyo Chemical Industry (Tokyo, Japan). Cedar Pollen Extract‐Cj was supplied by Cosmo Bio (Tokyo, Japan), and Imject Alum from Thermo Scientific (Waltham, MA). Cry j 1 and Biotin‐conjugated Cry j 1 were obtained from Hayashibara (Okayama, Japan). Histamine dihydrochloride was provided by Nacalai Tesque (Kyoto, Japan). Yucatan micropig skin was purchased from Charles River (Burlington, MA). A surfactant sucrose laurate (L‐195) was kindly supplied by Mitsubishi‐Kagaku Foods (Tokyo, Japan).

## 3 Methods

### 3.1 Preparation of S/O nanodispersions

The peptide 7CrpR (GIIAAYQNPASWKRRRSMKVTVAFNQFGPRRRDIFASKNFHLQKN RRRKLTSGKIASCLNRRRYGLVHVANNNYDPRRRSGKYEGGNIYTKKEAFNVERRRQFAKLTGFTLMG, underlined amino acids are arginine linkers) was produced in *Escherichia coli* and purified following our previously described method.^25^ A solution of the peptide in Milli‐Q water was stored at 4 °C until use. A water in oil (W/O) emulsion was prepared from an aqueous solution of 7CrpR (0.5 mg/ml, 2 ml) with or without R848 (0.25 mg/ml) and a cyclohexane solution of L‐195 (12.5 mg/ml, 4 ml) using a polytron homogenizer PT2500E (Kinematica AG, Luzern, Switzerland). The W/O emulsion was flash‐frozen in liquid nitrogen and the water and cyclohexane were removed by lyophilization for 24 h with a lyophilizer FDU‐1200 (Eyela, Tokyo, Japan). The resulting solid paste was dispersed in IPM (1 ml) to yield a S/O nanodispersion containing 1 mg/ml 7CrpR. Alternatively, the surfactant‐protein complex was dispersed in IPM (1 ml) containing R848 (0.5 mg/ml) to prepare a S/O nanodispersion containing R848 on the surface of the particles. Labeled S/O nanodispersions were prepare from 7CrpR labeled with Cy3 using a kit from GE healthcare (Buckinghamshire, UK).

The size distributions of the S/O nanodispersions were analyzed using a Zetasizer Nano ZS light scattering instrument (Malvern, Worcestershire, UK).

### 3.2 Drug release test

The drug release test was performed using custom‐fabricated Franz‐type diffusion cells with an effective diffusion area of 0.785 cm^2^ and a receptor volume of 5 ml. A polycarbonate film (Whatman Nuclepore Track‐Etch Membrane, 0.1 μm; GE healthcare) was set on a cell, and the receptor compartment filled with a phosphate buffered saline (PBS) solution containing 1% sodium dodecyl sulfate. A S/O nanodispersion (200 μl) was placed on the membrane and the cell was incubated for 48 h at 37 °C. Samples (200 μl) were extracted from the receptor compartment at 0, 3, 6, 24, and 48 h, and replaced with the same volume of fresh media. The peptide concentration was measured with a fluorescence spectrometer LS‐55 (PerkinElmer, Waltham, MA) at 540 nm (ex)/570 nm (em). R848 concentration in the receptor chamber was analyzed by HPLC and UV absorption (320 nm) on a Inertsil ODS‐3 C18, 5 μm, 4.6 × 250 mm column (GL Science, Tokyo, Japan), with a linear gradient from 95% water containing 0.1% TFA to 90% acetonitrile containing 0.1% TFA over 40 min (flow rate: 1.0 ml/min).

### 3.3 Histology

S/O nanodispersions containing 5‐CF‐7crpR (1 mg/ml in IPM) and Rho‐DOPE (50 μg/ml in IPM) were prepared as previously described.^29^ Mouse ear auricles were collected from ddY mice (7‐week‐old, female, Kyudo) and stored at −80 °C until use. Tissue papers impregnated with S/O nanodispersions (25 μl) were placed onto the dorsal skin of defrosted mouse auricles, tightly sealed in place with adhesive tape to model occlusive patches and incubated at 32 °C for 24 h. After removal of patches, the ear pieces were washed thoroughly with 99% ethanol followed by Milli‐Q water, and placed onto glass slides. Fluorescence images were obtained with a confocal laser scanning microscope LSM700 (Carl Zeiss, Oberkochen, Germany), by excitation at 488 nm (5‐CF) and at 555 nm (Rhodamine). A series of Z sectioning images was obtained at 5 μm intervals. The images showing 5‐CF (green) and Rhodamine (red) were exported as separate jpeg files (8‐bit RGB format, 512 pixels × 512 pixels, each). The RGB pixel values of green and red images were converted to brightness values (G and R, respectively), using a software ImageJ without any image processing.

### 3.4 Sensitization and immunotherapy

Mice were sensitized to Cj pollen according to our previous report.^25^ The Cj pollen extract was dissolved in PBS (100 μg/ml). Cj pollen extract in PBS (100 μl) and Imject Alum (100 μl) were mixed for 30 min and administered to mice by s.c. injection once a week for 3 weeks. Six days after the final s.c. injection, histamine dihydrochloride in PBS solution (2 μg/ml) was dropped into each nostril (5 μl each). The Cj pollen extract dissolved in PBS was challenged into each nostril (5 μl each) for 5 days from the day after the histamine administration. Blood samples were collected 3 days after the final challenge and total IgE levels in sera were measured to eliminate the mice having relatively mild pollen allergy. Three fourth of the mice showing higher serum total IgE levels were further subjected to immunotherapeutic treatment.

Patches carrying 7CrpR in PBS, and S/O nanodispersion with or without R848 were put onto the dorsal auricles of allergy model mice (25 μg 7CrpR each) once a week for 3 weeks (*n* = 6). The patches were applied for 48 h then removed. 7CrpR in a PBS solution was subcutaneously injected at the base of an auricle (50 μg) on the same dates as the patch administration for a positive control. Six days after the final peptide administration, histamine dihydrochloride in the PBS solution (2 μg/ml) was dropped into each nostril (5 μl each). The Cj pollen extract in PBS was challenged into each nostril (5 μl each) for 5 days from the day after histamine administration. Three days after the final challenge, blood samples were collected and incubated for 30 min at room temperature. The sera was obtained by centrifugation of the blood samples for 20 min at 800 × *g* and 4 °C, and antibody levels were measured.

### 3.5 Determination of serum antibody levels

Cry j 1‐specific IgG2a levels in the sera were measured by a standard ELISA. Briefly, a MaxiSorp 96‐well plate was coated with Cry j 1 (5 μg/ml, 100 μl) at 4 °C overnight. After washing and blocking, a 1:1,000 dilution of sera (100 μl) was added to each well, then incubated at 37 °C for 2 h. After washing, a solution of HRP‐conjugated anti mouse IgG2a (0.1 μg/ml, 100 μl) was added to each well and incubated at 37 °C for 1 h. After washing, color was developed in TMB solution (100 μl, eBioscience, San Diego, CA) at room temperature, the reaction was stopped after 20 min with 1 M sulfuric acid (100 μl). Absorbance at 450 nm adjusted with absorbance at 570 nm was read using a microplate reader Power Wave X (BioTek, Winooski, VT). Serum total IgE and Cry j 1‐specific IgE levels were measured by ELISA using a kit Mouse IgE Ready‐SET‐Go! (eBioscience) as previously described.^25^

A standard serum sample was obtained from a mouse subjected to s.c. injection of a mixture of Cj pollen extract and ImjectAlum once a week for 8 weeks. Each level of Cry j 1‐specific IgE and IgG2a in undiluted standard serum was assigned the arbitrarily value of 10,000 relative units (RU)/ml. The results are expressed as the mean ± standard deviation (*n* = 6). A one‐way analysis of variance followed by Tukey's test for multiple comparison was used to determine the significance of the data (* *p* < .05 and ** *p* < .01) using Prism6 software (Graph Pad Software, La Jolla, CA).

## 4 Results

**Figure 2:**
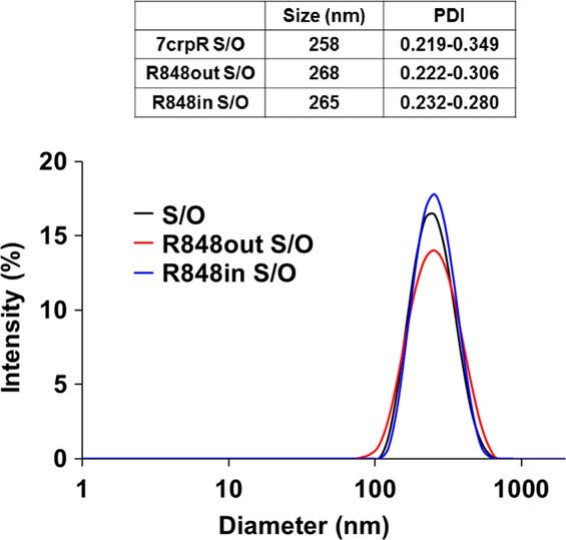
Size distribution of solid‐in‐oil nanodispersions containing the peptide 7CrpR alone (7CrpR), combined with R848 in the oil vehicle (R848out), or inside the nanoparticle (R848in)

**Figure 3:**
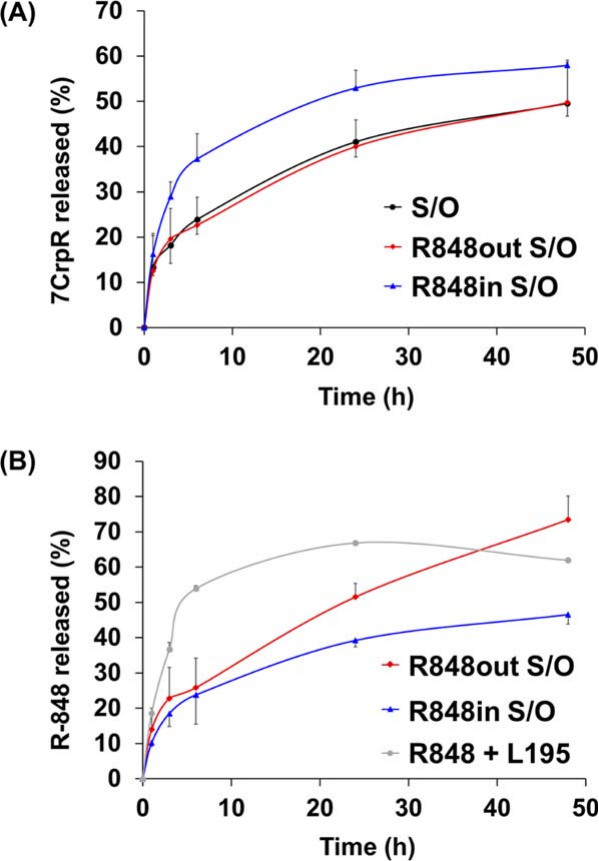
Release efficiencies of R848 (A) and 7CrpR (B) from solid‐in‐oil nanodispersions into aqueous media. Release of R848 from an IPM solution containing surfactant L195 was used as a control. Each point represents the mean ± SD of three duplicates. The data express the percentages of the cumulative amounts of bioactives in the receptor chambers

A S/O nanodispersion containing the peptide 7CrpR was prepared as previously described, with the average particle size of approximately 260 nm. R848 was soluble both in Milli‐Q water containing 5% ethanol and in IPM. We prepared S/O nanodispersions containing 0.5 mg/ml of R848 inside (R848in S/O) or outside (R848out S/O) the particles. The S/O nanodispersions containing 7CrpR and R848 were transparent. Dynamic light scattering (DLS) indicated that all the S/O nanodispersions showed single peaks, and the particle sizes and polydispersity indexes are shown in Figure 2. We first examined the release behavior of both peptide 7CrpR and R848 from the S/O nanodispersions using Franz‐type diffusion cells with polycarbonate membranes with 100 nm pores. The release profiles of 7CrpR and R848 are shown in Figure 3. The release efficiencies of 7CrpR were not affected by the presence of R848 in IPM (R848out S/O). The R848in S/O nanodispersion system showed slightly higher peptide release efficiency (Figure 3A). A burst release of R848 directly dissolved in IPM containing L‐195 (no S/O particles) was observed within the first 6 h (Figure 3B), and the release efficiency was sustained at approximately 60% until 48 h. R848in S/O particles demonstrated a gradual release curve for R848 that corresponded to that of 7CrpR. A gradual release of R848 from R848out S/O was also observed. However, the release efficiency was higher than that of R848in S/O.

**Figure 4:**
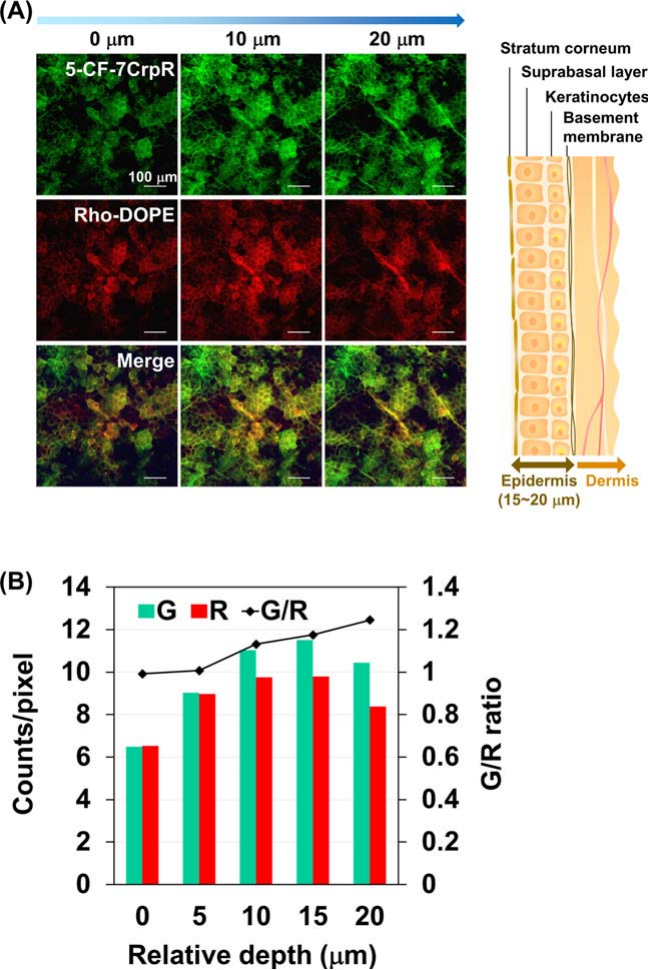
Histological analysis of mouse ear skin subjected to the labeled solid‐in‐oil nanodisperison. Confocal images are of the mouse ears subjected to the solid‐in‐oil nanodispersions containing 5‐carboxyfluorescein‐labeled 7CrpR (5‐CF‐7CrpR) and Rhodamine‐labeled dioleyl phosphatidylethanolamine (Rho‐DOPE) at 32°C for 24 h, and a schematic image of the mouse ear skin (A). The average brightness values of green (5‐CF) and red (Rhodamine) signals in the z‐sectioning images were quantified using the software ImageJ (B)

Transcutaneous permeation pathways of the peptide and the surfactant were examined next using 5‐CF‐labeled 7CrpR and Rhodamine‐labeled DOPE. The confocal fluorescence images of mouse ears subjected to the S/O nanodispersion carrying 5‐CF‐7CrpR and Rho‐DOPE for 24 h are shown in Figure 4. The green and red fluorescence signals from 5‐CF and Rhodamine, respectively, were observed at intercellular spaces. The mouse ear epidermis is composed of stratum corneum, suprabasal layer, and keratinocytes, and has an average thickness of 15–20 μm.^30^, ^31^ The red fluorescence from Rhodamine was observed at intercellular spaces near the skin surface, and the signal became weaker at depths greater than 10 μm. Conversely, the green fluorescence signal from 5‐CF was more clearly observed in the suprabasal layer and keratinocytes. The ratio of green to red fluorescence signals in the images increased with depth into the skin, indicating that the surfactant and the peptide had different permeabilities in the skin.

**Figure 5:**
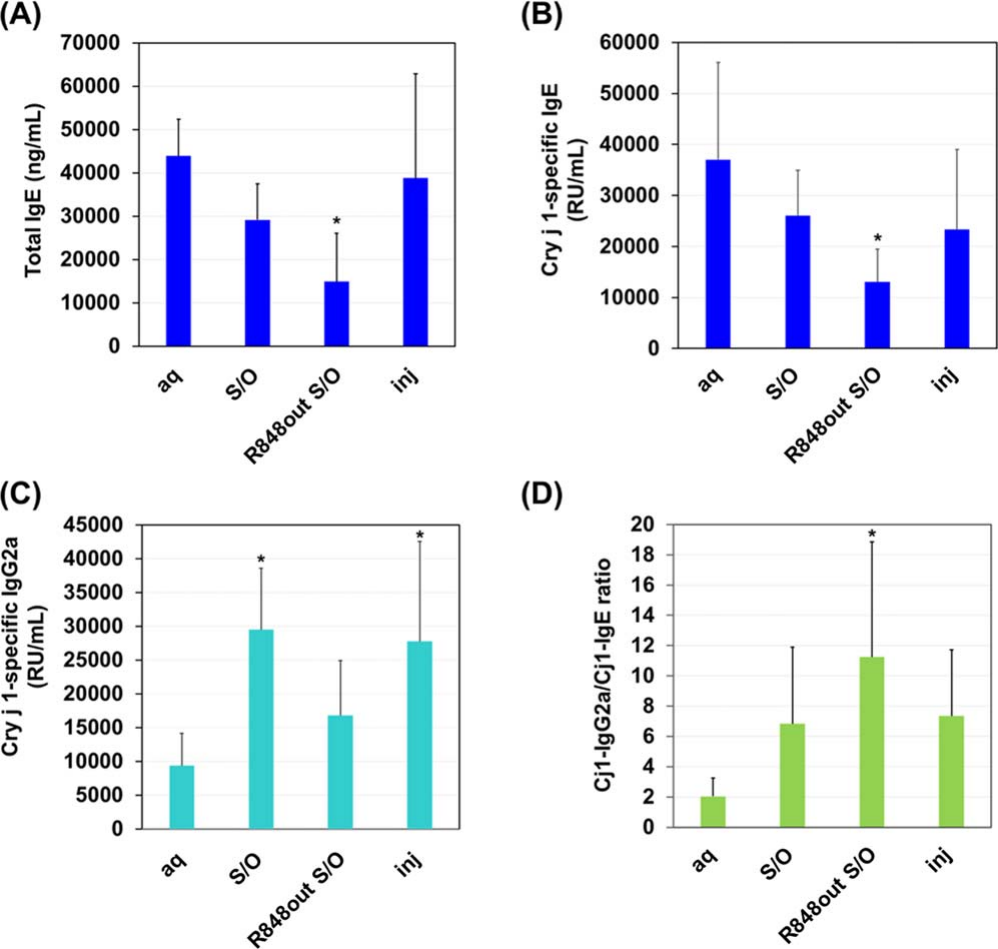
Serum antibody responses after immunotherapy using 7CrpR with or without R848 in the pollinosis model mice. The solid‐in‐oil nanodispersion system was administered once a week for 3 weeks, and the serum levels of total IgE (A), Cry j 1‐specific IgE (B) and Cry j 1‐specific IgG2a (C) antibodies were measured by ELISA. The ratios of Cry j 1‐specific IgG2a to Cry j 1‐specific IgE were calculated (D). The data represent the mean ± SD (*n* = 6). *p < .05

The mice showing 20 μg/ml and more total IgE values in sera after the first pollen challenge were considered to have pollen allergy. The R848out S/O system was applied to treat the pollinosis model mice. 7CrpR in PBS solutions were administered by s.c. injection or via a patch for control experiments. After treatment with 7CrpR in S/O nanodispersion system, both total IgE and Cry j 1‐specific IgE levels in sera declined compared with those from mice subjected to the patches carrying naked 7CrpR (Figure 5A,B). The peptide 7CrpR administered with S/O nanodispersion system or s.c. injection demonstrated corresponding efficacies in lowering IgE antibody levels. The total and antigen specific IgE levels significantly (*p* < .05) decreased with the S/O nanodispersion system carrying R848. In contrast to the decrease of IgE antibodies, Cry j 1‐specific IgG2a levels—an indicator of activation of Th1 cells—was greater with administration of 7CrpR via a patch or s.c. injection (Figure 5C). The ratio of Cry j 1‐specific IgG2a to Cry j 1‐specific IgE levels increased by administration of 7CrpR and R848 with the S/O nanodispersion system (Figure 5D).

## 5 Discussion

The peptide 7CrpR comprises seven human T cell determinants derived from two antigen molecules found in cedar pollen.^32^ Tri‐arginine linkers were used between each determinant of the 7CrpR, ensuring it dissolved in Milli‐Q water but not in the organic solvent IPM without encapsulation in the S/O nanoparticles. We have previously reported that the in vitro skin permeation efficiency of 7CrpR increased fourfold by applying the S/O nanodispersion system compared with that of naked 7CrpR dissolved in PBS.^24^ However, only a slight decrease of the IgE level was observed after administration of the peptide alone. In the present study, we introduced the adjuvant R848 to the S/O nanodispersion system. R848 is a small (Mw 314.38), imidazoquinoline compound, and is a known TLR 7/8 agonist that efficiently alleviates allergic symptoms by modulating the balance of immune responses in the body.^32^ R848 was expected to permeate through the skin efficiently because of its size and moderate hydrophobicity. DLS analysis indicated that the inclusion of R848 to the S/O particles had negligible effect on particle sizes, regardless of whether it was added into the particle or in the oil vehicle. In vitro peptide release efficiencies from the S/O nanodispersions were not affected by the presence of R848. The release behavior of R848 varied depending on the location of the R848. R848 dissolved in IPM containing surfactant L‐195 was released rapidly into aqueous media and the release ratio reached a saturation point of around 60% after 6 h, probably because the distribution ratio of R848 between the receptor phase and the donor phase was approximately 6:4. R848 encapsulated within the particle revealed a gradual release curve similar to that of the 7CrpR, indicating that the release efficiency of R848 into the aqueous medium was dominated by the collapse efficiency of the S/O nanoparticles. R848 adhered to the outside of the particles was also released gradually. The results suggested that R848 molecules interacted with the S/O particles in the beginning, with the interaction becoming weaker when the S/O particles collapsed. These results reveal that the S/O nanodispersion system provides gradual and long‐lasting release of R848. We chose to use R848out S/O for immunotherapy of pollinosis in the model mice.

We next observed the skin permeation pathways of the peptides and surfactants. 5‐CF‐FITC was encapsulated in the S/O nanoparticles, and 0.1% of L‐195 was substituted with Rho‐DOPE. The confocal fluorescence microscopic images of mouse ears subjected to the labeled S/O nanodispersion for 24 h revealed that the S/O nanodispersion permeated via the intercellular pathway. The brightness of Z sectioning images indicated that 5‐CF‐7CrpR permeated more deeply than the Rho‐DOPE. This result agrees well with our previous result that indicated that most of the S/O nanoparticles collapsed in stratum corneum, and only the released protein molecules permeated into the hydrophilic viable epidermis and dermis in the Yucatan micropig skin.^33^ Yellow dots observed in the merge images suggested aggregation of the peptides and the surfactants, which was not observed in the images of mouse ears subjected to the S/O nanodispersions of ovalbumin.^29^ Large aggregates of the S/O components were supposed to have formed after collapse of the nanoparticles because 7CrpR does not have a specific conformation and 7CrpR is composed of approximately 30% hydrophobic amino acids. Introduction of longer arginine linkers may prevent formation of large aggregates and enhance the permeation efficiency of the peptide. Despite the formation of large aggregates, the fluorescence microscopic images demonstrated that some 5‐CF‐7CrpR permeated through the stratum corneum.

Type I allergies, such as pollinosis, display the distinctive symptom of enhanced production of IgE antibodies in sera,^34^ and the serum total IgE level is often used as a marker of possible allergy. An antigen‐specific IgE level could be used to assess whether or not the patient has an allergy to the specific antigen. In our experiments, unprimed mice produced undetectable levels of total IgE in sera. The mice sensitized to the Cj pollen extract produced more than 20 μg/ml of total IgE. After the treatment of the pollinosis model mice with the S/O nanodispersions, the total IgE and the Cry j 1‐specific IgE levels in the sera declined, even without R848. The decrease in IgE levels was caused by efficient delivery of 7CrpR to the viable epidermis beneath the stratum corneum using the S/O nanodispersion system. The total and antigen specific IgE levels significantly decreased with the addition of R848, indicating alleviation of allergy.

R848 is recognized by TLR7 and TLR8 in the humans, and by TLR7 in mice, and is known to activate immune cells via the MyD88‐dependent signaling pathway.^35^ Several reports indicated that R848 had the ability to skew the Th1/Th2 balance to Th1‐type immunity through the activation of Th1‐related lymphocytes.^36^, ^37^ Secretion of IL‐12, induction of IgG2a and reduction of IgE had been reported after administration of R848,^38^, ^39^, ^40^ while T cell epitope vaccine itself also enhances the production of IgG subclass antibodies that are related to the Th1‐type immune response.^12^ Therefore, we examined the antibody responses in the sera of mice that had been subjected to S/O and R848out S/O nanodispersions. Increases in the IgG2a antibody levels were observed in all the sera collected from the mice treated by S/O patch or s.c. injection, regardless of the peptide administration method. The ratio of Cry j 1‐specific IgG2a to Cry j 1‐specific IgE levels indicated that the immune response was biased to Th1‐type immunity in the mice subjected to 7CrpR. Moreover, the bias of the immune response to the Th1‐type immunity was enhanced by the addition of R848. Overall antibody levels decreased in the presence of R848, indicating that the immune response itself might be inhibited by the transcutaneous administration of the adjuvant. These results indicated that both hydrophilic 7CrpR and hydrophobic R848 were efficiently delivered through the skin.

## 6 Conclusions

We have demonstrated the potency of the S/O nanodispersion system for the transcutaneous immunotherapy of Japanese cedar pollinosis. The peptide vaccine was delivered to the viable epidermis through the stratum corneum. The S/O nanodispersion system was able to carry both a hydrophilic peptide vaccine and a hydrophobic adjuvant simultaneously. Transcutaneously administered T cell epitope peptide in combination with the hydrophobic adjuvant R848 using the S/O nanodispersion system efficiently lowered antigen‐specific IgE levels, and shifted the Th1/Th2 immune balance in the pollinosis model mice toward Th1‐type immunity.

## Conflict of interest

The authors declare no conflict of interest.

## References

[1] Stern AM , Markel H. The history of vaccines and immunization: familiar patterns, new challenges. Health Aff. (Millwood) 2005;24:611–621. 15886151 10.1377/hlthaff.24.3.611

[2] Larché M , Wraith DC. Peptide‐based therapeutic vaccines for allergic and autoimmune diseases. Nat Med. 2005;11:S69–S76. 15812493 10.1038/nm1226

[3] Li W , Joshi MD , Singhania S , Ramsey KH , Murthy AK. Peptide vaccine: progress and challenges. Vaccines (Basel). 2014;2:515–536. 26344743 10.3390/vaccines2030515 PMC4494216

[4] El‐Qutob D , Reche P , Subiza JL , Fernández‐Caldas E. Peptide‐based allergen specific immunotherapy for the treatment of allergic disorders. IAD. 2015;9:16–22. 10.2174/1872213x09666150302105555 25760734

[5] Coombs RRA , Gell PGH. Classification of allergic reactions responsible for clinical hypersensitivity and disease In: Gell PGH, Coombs RRA, eds. Clinical Aspects of Immunology. 2nd ed. Oxford and Edinburgh: Blackwell Scientific Publications; 1968:575–596.

[6] Descotes J , Choquet‐Kastylevsky G. Gell and Coombs's classification: is it still valid? Toxicology. 2001;158:43–49. 11164991 10.1016/s0300-483x(00)00400-5

[7] Rajan TV. The Gell‐Coombs classification of hypersensitivity reactions: a re‐interpretation. Trends Immunol. 2003;24:376–379. 12860528 10.1016/s1471-4906(03)00142-x

[8] Peavy RD , Metcalfe DD. Understanding the mechanisms of anaphylaxis. Curr Opin Allergy Clin Immunol. 2008;8:310–315. 18596587 10.1097/ACI.0b013e3283036a90 PMC2683407

[9] Akdis CA , Blaser K. Mechanisms of allergen‐specific immunotherapy. Allergy. 2000;55:522–530. 10858982 10.1034/j.1398-9995.2000.00120.x

[10] Valenta R. The future of antigen‐specific immunotherapy of allergy. Nat Rev Immunol. 2002;2:446–453. 12093011 10.1038/nri824

[11] Akdis CA , Akdis M. Mechanisms of allergen‐specific immunotherapy and immune tolerance to allergens. World Allergy Organ J. 2015;8:17 26023323 10.1186/s40413-015-0063-2 PMC4430874

[12] Prickett SR , Rolland JM , O'Hehir RE. Immunoregulatory T cell epitope peptides: the new frontier in allergy therapy. Clin Exp Allergy. 2015;45:1015–1026. 25900315 10.1111/cea.12554 PMC4654246

[13] O'Hehir RE , Prickett SR , Rolland JM. T cell epitope peptide therapy for allergic diseases. Curr Allergy Asthma Rep. 2016;16:14. 26768622 10.1007/s11882-015-0587-0 PMC4713452

[14] Takaiwa F , Yang L. Development of a rice‐based peptide vaccine for Japanese cedar and cypress pollen allergies. Transgenic Res. 2014;23:573–584. 24638148 10.1007/s11248-014-9790-3

[15] Kawabe Y , Hayashida Y , Numata K , et al. Oral immunotherapy for pollen allergy using T‐cell epitope‐containing egg white derived from genetically manipulated chickens. PLoS One. 2012;7:e48512 23144766 10.1371/journal.pone.0048512 PMC3483267

[16] Yamada T , Saito H , Fujieda S. Present state of Japanese cedar pollinosis: the national affliction. J Allergy Clin Immunol. 2014;133:632–629. 24361081 10.1016/j.jaci.2013.11.002

[17] Okamoto Y , Horiguchi S , Yamamoto H , Yonekura S , Hanazawa T. Present situation of cedar pollinosis in Japan and its immune responses. Allergol Int. 2009;58:155–162. 19307773 10.2332/allergolint.08-RAI-0074

[18] Mitragotri S. Immunization without needles. Nat Rev Immunol. 2005;5:905–916. 16239901 10.1038/nri1728

[19] Mueller SN , Zaid A , Carbone FR. Tissue‐resident T cells: dynamic players in skin immunity. Front Immunol. 2014;5:332 25076947 10.3389/fimmu.2014.00332 PMC4099935

[20] Heath WR , Carbone FR. The skin‐resident and migratory immune system in steady state and memory: innate lymphocytes, dendritic cells and T cells. Nat Immunol. 2013;14:978–985. 24048119 10.1038/ni.2680

[21] Tay SS , Roediger B , Tong PL , Tikoo S , Weninger W. The skin‐resident immune network. Curr Dermatol Rep. 2013;3:13–22. 24587975 10.1007/s13671-013-0063-9 PMC3931970

[22] Zaffaroni A. Alza: an enterprise in biomedical innovation. Technovation. 1981;1:135–146.

[23] Bal SM , Ding Z , van Riet E , Jiskoot W , Bouwstra JA. Advances in transcutaneous vaccine delivery: do all ways lead to Rome? J Control Release. 2010;148:266–282. − 20869998 10.1016/j.jconrel.2010.09.018

[24] Matsuo K , Hirobe S , Okada N , Nakagawa S. Frontiers of transcutaneous vaccination systems: novel technologies and devices for vaccine delivery. Vaccine. 2013;31:2403–2415. 23523401 10.1016/j.vaccine.2013.03.022 PMC7125630

[25] Kitaoka M , Shin Y , Kamiya N , Kawabe Y , Kamihira M , Goto M. Transcutaneous peptide immunotherapy of Japanese cedar pollinosis using solid‐in‐oil nanodispersion technology. AAPS PharmSciTech. 2015;16:1418–1424. 25986596 10.1208/s12249-015-0333-x PMC4666251

[26] Tahara Y , Kamiya N , Goto M. Solid‐in‐oil dispersion: a novel core technology for drug delivery systems. Int J Pharm. 2012;438:249–257. 22975308 10.1016/j.ijpharm.2012.09.007

[27] Martins M , Loureiro A , Azoia NG , Silva C , Cavaco‐Paulo A. Protein formulations for emulsions and solid‐in‐oil dispersions. Trends Biotechnol. 2016;34:496–505. 26996614 10.1016/j.tibtech.2016.03.001

[28] Quarcoo D , Weixler S , Joachim RA , et al. Resiquimod, a new immune response modifier from the family of imidazoquinolinamines, inhibits allergen‐induced Th2 responses, airway inflammation and airway hyper‐reactivity in mice. Clin Exp Allergy. 2004;34:1314–1320. 15298575 10.1111/j.1365-2222.2004.02023.x

[29] Kitaoka M , Imamura K , Hirakawa Y , Tahara Y , Kamiya N , Goto M. Sucrose laurate‐enhanced transcutaneous immunization with a solid‐in‐oil nanodispersion. Med Chem Commun. 2014;5:20–24.

[30] Mulholland WJ , Arbuthnott EA , Bellhouse BJ , et al. Multiphoton high‐resolution 3D imaging of Langerhans cells and keratinocytes in the mouse skin model adopted for epidermal powdered immunization. J Invest Dermatol. 2006;126:1541–1548. 16645596 10.1038/sj.jid.5700290

[31] Li JL , Goh CC , Keeble JL , et al. Intravital multiphoton imaging of immune responses in the mouse ear skin. Nat Protoc. 2012;7:221–234. 22240584 10.1038/nprot.2011.438

[32] Hirahara K , Tatsuta T , Takatori T , et al. Preclinical evaluation of an immunotherapeutic peptide comprising 7 T‐cell determinants of Cry j 1 and Cry j 2, the major Japanese cedar pollen allergens. J Allergy Clin Immunol. 2001;108:94–100. 11447388 10.1067/mai.2001.115481

[33] Tahara Y , Honda S , Kamiya N , et al. A solid‐in‐oil nanodispersion for transcutaneous protein delivery. J Control Release. 2008;131:14–18. 18687370 10.1016/j.jconrel.2008.07.015

[34] Ribatti D. The discovery of immunoglobulin E. Immunol Lett. 2016;171:1–4. 26772434 10.1016/j.imlet.2016.01.001

[35] Hemmi H , Kaisho T , Takeuchi O , et al. Small anti‐viral compounds activate immune cells via the TLR7 MyD88‐dependent signaling pathway. Nat Immunol. 2002;3:196–200. 11812998 10.1038/ni758

[36] Brugnolo F , Sampognaro S , Liotta F , et al. The novel synthetic immune response modifier R‐848 (Resiquimod) shifts human allergen‐specific CD4+ TH2 lymphocytes into IFN‐gamma‐producing cells. J Allergy Clin Immunol. 2003;111:380–388. 12589360 10.1067/mai.2003.102

[37] Grela F , Aumeunier A , Bardel E , et al. The TLR7 agonist R848 alleviates allergic inflammation by targeting invariant NKT cells to produce IFN‐gamma. J Immunol. 2011;186:284–290. 21131420 10.4049/jimmunol.1001348

[38] Shen E , Lu L , Wu C. TLR7/8 ligand, R‐848, inhibits IgE synthesis by acting directly on B lymphocytes. Scand J Immunol. 2008;67:560–568. 18397197 10.1111/j.1365-3083.2008.02105.x

[39] Vasilakos JP , Smith RM , Gibson SJ , et al. Adjuvant activities of immune response modifier R‐848: comparison with CpG ODN. Cell Immunol. 2000;204:64–74. 11006019 10.1006/cimm.2000.1689

[40] Siebeneicher S , Reuter S , Krause M , et al. Epicutaneous immune modulation with Bet v 1 plus R848 suppresses allergic asthma in a murine model. Allergy. 2014;69:328–337. 24329861 10.1111/all.12326

[41] Vasilakos JP , Tomai MA. The use of Toll‐like receptor 7/8 agonists as vaccine adjuvants. Expert Rev Vaccines. 2013;12:809–819. 23885825 10.1586/14760584.2013.811208

